# Risk of acute myocardial infarction in patients with gastroesophageal reflux disease: A nationwide population-based study

**DOI:** 10.1371/journal.pone.0173899

**Published:** 2017-03-20

**Authors:** Wei-Yi Lei, Jen-Hung Wang, Shu-Hui Wen, Chih-Hsun Yi, Jui-Sheng Hung, Tso-Tsai Liu, William C. Orr, Chien-Lin Chen

**Affiliations:** 1 Department of Medicine, Hualien Tzu Chi Hospital, Buddhist Tzu Chi Medical Foundation and Tzu Chi University, Hualien, Taiwan; 2 Institute of Medical Sciences, Tzu Chi University, Hualien, Taiwan; 3 Department of Medical Research, Hualien Tzu Chi Hospital, Buddhist Tzu Chi Medical Foundation, Hualien, Taiwan; 4 Department of Public Health Tzu Chi University, Hualien, Taiwan; 5 Lynn Institute for Healthcare Research, University of Oklahoma Health Sciences Center, Oklahoma City, Oklahoma, United States of America; Universitatsklinikum Freiburg, GERMANY

## Abstract

**Objective:**

Gastroesophageal reflux disease (GERD) is a common disease which can cause troublesome symptoms and affect quality of life. In addition to esophageal complications, GERD may also be a risk factor for extra-esophageal complications. Both GERD and coronary artery disease (CAD) can cause chest pain and frequently co-exist. However, the association between GERD and acute myocardial infarction (AMI) remain unclear. The purpose of the study was to compare the incidence of acute myocardial infarction in GERD patients with an age-, gender-, and comorbidity matched population free of GERD. We also examine the association of the risk of AMI and the use of acid suppressing agents in GERD patients.

**Methods:**

We identified patients with GERD from the Taiwan National Health Insurance Research Database. The study cohort comprised 54,422 newly diagnosed GERD patients; 269,572 randomly selected age-, gender-, comorbidity-matched subjects comprised the comparison cohort. Patients with any prior CAD, AMI or peripheral arterial disease were excluded. Incidence of new AMI was studied in both groups.

**Results:**

A total 1,236 (0.5%) of the patients from the control group and 371 (0.7%) patients from the GERD group experienced AMI during a mean follow-up period of 3.3 years. Based on Cox proportional-hazard model analysis, GERD was independently associated with increased risk of developing AMI (hazard ratio (HR) = 1.48; 95% confidence interval (CI): 1.31–1.66, *P* < 0.001). Within the GERD group, patients who were prescribed proton pump inhibitors (PPIs) for more than one year had slightly decreased the risk of developing AMI, compared with those without taking PPIs (HR = 0.57; 95% CI: 0.31–1.04, *P* = 0.066).

**Conclusions:**

This large population-based study demonstrates an association between GERD and future development of AMI, however, PPIs use only achieved marginal significance in reducing the occurrence of AMI in GERD patients. Further prospective studies are needed to evaluate whether anti-reflux medication may reduce the occurrence of acute ischemic event in GERD patients.

## Introduction

Gastroesophageal reflux disease (GERD) is a common disorder which can cause troublesome reflux symptoms and potential serious complications, and has a negative impact on the quality of life [1]. In addition to esophageal complications, GERD may also be a risk factor for extra-esophageal complications including laryngeal, pulmonary, and cardiovascular diseases [2].

GERD more frequently causes chest pain than other esophageal motility disorders [3], implying that GERD symptoms may be easily misclassified as coronary artery disease (CAD). As both GERD and CAD are prevalent diseases in the population, they frequently co-exist; hence frequently making a differential diagnosis of chest pain more difficult. In addition, the distal esophagus and the heart have overlapping sensory pathways and share a common afferent vagal supply [4], suggesting the notion that location and radiation of perceived pain may be identical. Therefore, evaluating the symptoms is not sufficient to diagnose the underlying disease.

In 1962, Smith and Papp introduced the term “linked angina”, which implies that esophageal dysfunction can trigger myocardial ischemia [5]. Chauhan et al have shown that esophageal acid stimulation can significantly reduce coronary blood flow and produce angina in patients with syndrome X and CAD. This phenomenon was absent in the heart transplant recipients, in whom the heart was denervated, supporting that reduced coronary blood flow was accomplished through a cardioesophageal reflex [6, 7]. Previous studies have demonstrated that GERD is common in patients with CAD [8, 9]. One prospective case-control study based on the UK General Practice Research Database also showed an association between GERD and angina pectoris [10]. However, the association between GERD and acute myocardial infarction (AMI) remains undetermined.

The aim of this study was to assess the incidence of AMI in GERD patients and to compare it with general population free of GERD. We also investigate the association between the use of proton pump inhibitors (PPIs) and the risk of development of AMI in the cohort of GERD patients.

## Materials and methods

### Ethics statement

The protocol for the research project has been approved by Ethics Committee of Tzu Chi Medical Center (Taiwan) and it conforms to the provisions of the Declaration of Helsinki in 1995 (as revised in Edinburgh 2000). The informed written consent was obtained from each subject, and patient anonymity was preserved. The study was approved by the Research Ethics Committee of Hualien Tzu Chi Hospital, Buddhist Tzu Chi Medical Foundation, Hualien, Taiwan.

### Data source

The National Health Insurance (NHI) program in Taiwan was established in 1995, and covered over 23 million residents by 2010, which represented more than 99% of Taiwan’s population. The National Health Insurance Research Database (NHIRD) contained comprehensive health information including patients’ socio-demographic data, diagnostic codes based on the International Classification of Diseases, 9th revision, Clinical Modification (ICD-9-CM) codes, medical procedures, and drug prescriptions. The National Health Research Institute (NHRI) has released a cohort dataset composed of 1,000,000 randomly sampled people who were alive during 2000, and randomly sampled from the registry of all NHI enrollees. The study samples were retrieved from the Longitudinal Health Insurance Database 2000 (LHID 2000). The source data was encrypted to protect privacy and the data extracted was anonymous.

### Study design and participants

We conducted a retrospective cohort study of patients newly diagnosed with GERD (ICD-9-CM codes: 530.11 or 530.81) between January 1, 2000 and December 31, 2011. We enrolled 54,422 GERD subjects (older than 18 years) in LHID 2000. The Bureau of National Health Insurance requires that GERD patients be diagnosed by either endoscopy or 24-hour pH-meter inspection before PPIs can be prescribed for treatment. The criteria has been used in similar studies [11], thus the diagnoses of GERD are valid. For each patient in GERD cohort, 5 subjects without GERD matched for age, gender, comorbidities, index year via propensity score matching were included as the comparison cohort. Comorbidities included preexisting hypertension, diabetes mellitus, hyperlipidemia, ischemic stroke, and congestive heart failure. The outpatient pharmacy prescription database was used to identify the drug types, dosages, date of prescriptions, supply days, and total number of pills dispensed. Medication use of PPIs (ATC code: A02BC) corresponding to GERD cohort was summarized.

### Acute myocardial infarction event measurement

**Fig 1:**
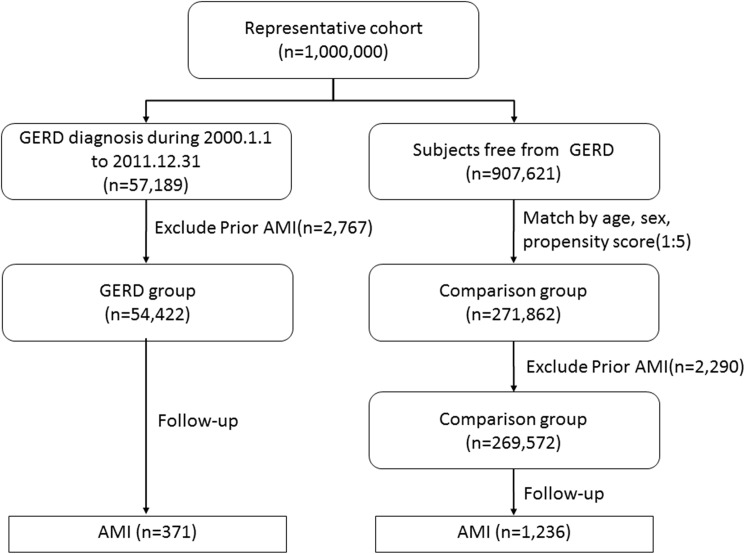
Flowchart of subjects enrolled into the study.

AMI was identified using the ICD-9-CM code: 410.XX. ST elevation myocardial infarction (STEMI) was identified using the ICD-9-CM code: 410.0–410.6 and non-ST elevation myocardial infarction (NSTEMI) was identified using the ICD-9-CM code: 410.7 or 410.9. Participants were followed from the index date until the earliest occurrence of AMI in hospitalization records or the date of death, dropout from the insurance program, or to the end of the study period. We excluded subjects who had been diagnosed with CAD or peripheral artery disease or AMI before enrollment. The flow chart for the selection process is shown in Fig 1.

### Statistical analysis

Chi-square and independent t tests were used to assess the between-cohort differences in frequencies or means of variables. Cohen's d was adopted to evaluate the effect size. Because the main interest of this study was GERD and the risk of AMI, each participant was followed to accumulate person-time beginning from the index date to the newly-onset AMI during a 12-year follow-up period. If patients died before the onset of AMI, they were censored to account for the competing risks attributable to other causes. All cases with no endpoint occurring during follow-up were also censored. After matching by age, gender, and propensity scores, the Kaplan-Meier curve and log-rank test were used to estimate and compare the incidence rates of hospitalizations for AMI. The Cox proportional hazard model was used to estimate the hazard ratio (HR) and the accompanying 95% confidence interval (CI) of AMI with adjustment for confounders. A significance level of 0.05 was considered statistically significant. The statistical software packages SAS (version 9.4; SAS Institute, Inc., Cary, NC, USA) and SPSS (version 17; SPSS Inc., Chicago, IL, USA) were used for data analysis.

## Results

### Participant characteristics

The demographic characteristics of the study subjects are shown in Table 1. The study identified 54,422 patients with newly diagnosed GERD (mean age = 51.63 years, standard deviation [SD] = 16.95) from the LHID 2000. Another 269,572 control subjects, matched for age and co-morbidities were enrolled as the control group. Most patients (75.1%) were aged more than 40 years, and the majority of patients in both cohorts were women (53.5%). The most common comorbidities were hypertension and diabetes mellitus. There were no statistically significant differences in the age, sex, and baseline comorbidities among the two groups.

**Table 1:** Demographic data on patients with and without gastroesophageal reflux disease.

Variables	GERD (n = 54,422)		Control (n = 269,572)		P-value	Cohen's d
**Gender**					0.635	
Male	25305	46.5%	125045	46.4%		
Female	29117	53.5%	144527	53.6%		
**Age**	51.63±16.95		51.47±16.90		0.039*	<0.01
**Age Group**					0.501	
<20 y/o	1164	2.1%	5820	2.2%		
20-30 y/o	4529	8.3%	22632	8.4%		
30-40 y/o	7883	14.5%	39388	14.6%		
40-50 y/o	11299	20.8%	56378	20.9%		
50-60 y/o	12331	22.7%	61276	22.7%		
≧60 y/o	17216	31.6%	84078	31.2%		
**Hypertension**	7394	13.6%	36412	13.5%	0.623	<0.01
**Diabetes**	2855	5.2%	13703	5.1%	0.116	<0.01
**Hyperlipidemia**	2605	4.8%	12185	4.5%	0.007*	0.01
**Congestive Heart Failure**	336	0.6%	1485	0.6%	0.058	<0.01
**Ischemic Stroke**	613	1.1%	2754	1.0%	0.029*	0.01
**Follow-up years (median)**	3.30(1.66–5.17)		3.31(1.67–5.18)		0.649	

Data are presented as n and percentage.

*P-value < 0.05 was considered statistically significant after test.

### Incidence of acute myocardial infarction

**Fig 2:**
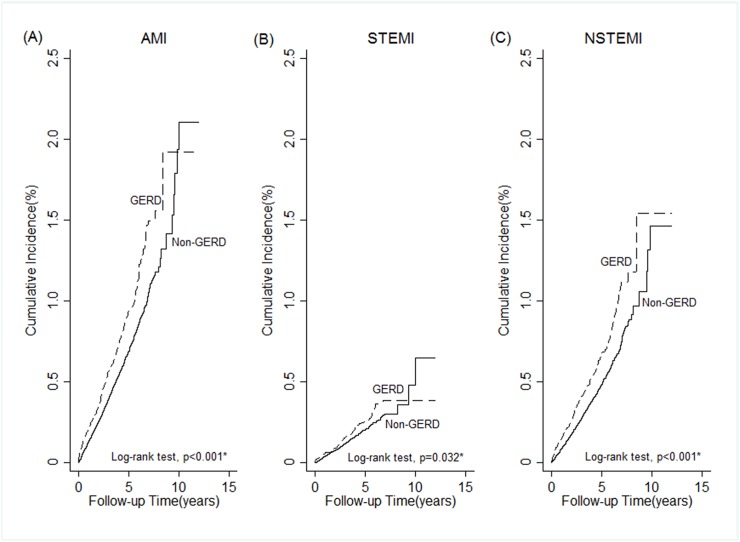
Kaplan-Meier curves showing a significant difference in cumulative incidence of acute myocardial infarction (AMI) among patients with GERD and controls.

**Fig 3:**
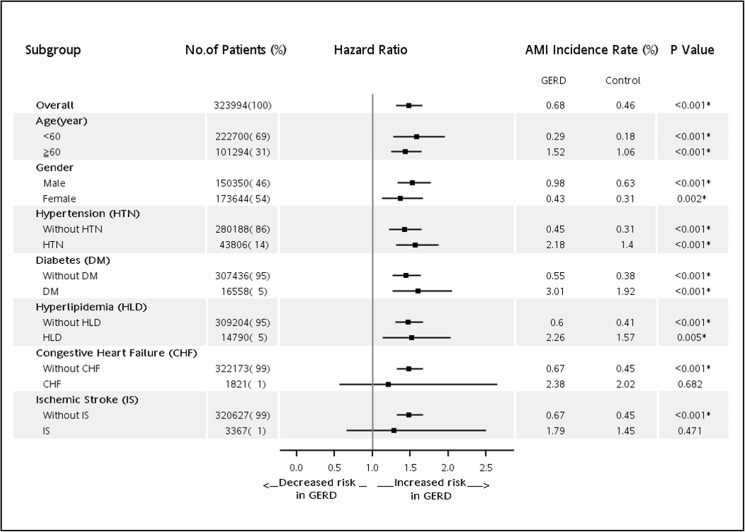
Subgroup analyses of new-onset acute myocardial infarction in patients with GERD.

Out of the 323,994 patients and controls, 1,607 (0.5%) were diagnosed as having AMI over a mean follow-up period of 3.3 years. Subjects aged over 40 years had a significantly higher probability of developing AMI than those aged less than 40 years. Men had a significantly higher probability of developing AMI than women (adjusted HR = 1.99, 95% CI: 1.80–2.21). Moreover, patients with comorbidities of hypertension, diabetes, hyperlipidemia, congestive heart failure and GERD had increased risk of AMI. Subjects with comorbidity of diabetes mellitus had the highest hazard (HR = 2.02, 95% CI: 1.78–2.30) of AMI than those had no diabetes (Table 2). A total of 371 (0.68%) patients from the GERD group and 1,236 (0.46%) of the controls developed AMI during the follow-up period. The log-rank test revealed that patients with GERD had significantly higher incidence of AMI than those without GERD (P < 0.001). The results of a Kaplan-Meier analysis are shown in Fig 2. We further classified the type of AMI into ST elevation myocardial infarction (STEMI, ICD9 codes: 410.0–410.6) and non-ST elevation myocardial infarction (NSTEMI, ICD9 codes: 410.7 or 410.9). Among the 1,607 patients of AMI, 458(29%) were STEMI and 1,149(71%) were NSTEMI. GERD was associated with a higher risk of AMI and NSTEMI. The association between GERD and STEMI was similar, but the result only showed marginal significance due to small number of STEMI (Table 2 and Fig 2). After Cox proportional-hazard model analysis, GERD was independently associated with increased risk of developing AMI (HR = 1.48; 95% CI: 1.31–1.66, P < 0.001, Table 3). Furthermore, subgroup analyses based on age, gender and comorbidities were conducted to evaluate the risk of AMI (Fig 3). Our subgroup analyses indicated that patients with GERD were significantly positively associated with the presence of AMI, except for patients in congestive heart failure subgroup (HR = 1.21; 95% CI: 0.56–2.65, P = 0.682) and ischemic stroke subgroup (HR = 1.28; 95% CI: 0.66–2.5, P = 0.471). We further investigated the patients according to whether or not PPIs were administered for GERD therapy. There were 1,345 patients who received PPIs for more than one year and 38,067 patients who did not received any PPIs. GERD patients who were prescribed PPIs for more than one year had a trend in lower risk of developing AMI, compared with those who were not taking PPIs (HR = 0.57; 95% CI: 0.31–1.04, P = 0.066) (Table 3).

**Table 2:** Independent predictors of new-onset acute myocardial infarction.

Variables	AMI			STEMI			NSTEMI		
	N (%)	AHR^a^ (95% CI)	P-value	N (%)	AHR^a^ (95% CI)	P-value	N (%)	AHR^a^ (95% CI)	P-value
**Age Group**									
<40 y/o	35 (0.04%)	1.00		20 (0.02%)	1.00		15 (0.02%)	1.00	
40-50 y/o	136 (0.20%)	4.38 (3.02–6.35)	<0.001*	56 (0.08%)	3.21 (1.92–5.35)	<0.001*	80 (0.12%)	5.80 (3.34–10.09)	<0.001*
50-60 y/o	281 (0.38%)	7.80 (5.48–11.10)	<0.001*	93 (0.13%)	4.88 (3.00–7.94)	<0.001*	188 (0.26%)	12.10 (7.14–20.49)	<0.001*
≧60 y/o	1155 (1.14%)	17.35 (12.34–24.40)	<0.001*	289 (0.29%)	9.89 (6.22–15.74)	<0.001*	866 (0.85%)	33.67 (20.12–56.35)	<0.001*
**Gender**									
Female	570 (0.33%)	1.00		119 (0.07%)	1.00		451 (0.26%)	1.00	
Male	1037 (0.69%)	1.99 (1.80–2.21)	<0.001*	339 (0.23%)	3.30 (2.68–4.07)	<0.001*	698 (0.46%)	1.80 (1.60–2.03)	<0.001*
**GERD**									
No	1236 (0.46%)	1.00		359 (0.13%)	1.00		877 (0.33%)	1.00	
Yes	371 (0.68%)	1.48 (1.31–1.66)	<0.001*	99 (0.18%)	1.22 (0.97–1.53)	0.086	272 (0.50%)	1.33 (1.15–1.53)	<0.001*
**Hypertension**									
No	938 (0.33%)	1.00		293 (0.10%)	1.00		645 (0.23%)	1.00	
Yes	669 (1.53%)	1.70 (1.52–1.89)	<0.001*	165 (0.38%)	1.70 (1.38–2.11)	<0.001*	504 (1.15%)	1.79 (1.57–2.03)	<0.001*
**Diabetes**									
No	1258 (0.41%)	1.00		387 (0.13%)	1.00		871 (0.28%)	1.00	
Yes	349 (2.11%)	2.02 (1.78–2.30)	<0.001*	71 (0.43%)	1.63 (1.24–2.15)	<0.001*	278 (1.68%)	2.48 (2.14–2.87)	<0.001*
**Hyperlipidemia**									
No	1357 (0.44%)	1.00		399 (0.13%)	1.00		958 (0.31%)	1.00	
Yes	250 (1.69%)	1.59 (1.38–1.83)	<0.001*	59 (0.40%)	1.43 (1.07–1.91)	0.017*	191 (1.29%)	1.48 (1.25–1.75)	<0.001*
**Congestive Heart Failure**									
No	1569 (0.49%)	1.00		451 (0.14%)	1.00		1118 (0.35%)	1.00	
Yes	38 (2.09%)	1.49 (1.08–2.06)	0.016*	7 (0.38%)	1.29 (0.61–2.73)	0.508	31 (1.70%)	1.90 (1.32–2.74)	0.001*
**Ischemic Stroke**									
No	1556 (0.49%)	1		445 (0.14%)	1		1111 (0.35%)	1	
Yes	51 (1.51%)	0.86 (0.65–1.14)	0.297	13 (0.39%)	1.04 (0.59–1.82)	0.899	38 (1.13%)	1.02 (0.73–1.41)	0.927

^a^ Cox's proportional hazards model; AMI, acute myocardial infarction; STEMI, ST elevation acute myocardial infarction; NSTEMI, Non-ST elevation acute myocardial infarction; AHR, adjusted hazard ratio; CI, confidence interval.

*P-value < 0.05 was considered statistically significant after test.

**Table 3:** Independent predictors of new-onset acute myocardial infarction in patients with gastroesophageal reflux disease under proton pump inhibitors use.

Variable	Crude HR (95% CI)	P-value	Adjusted HR (95% CI)	P-value
PPIs				
Non-GERD(n = 269,572) vs GERD without PPI(n = 38,067)	0.71(0.62, 0.81)	<0.001*	0.69(0.60, 0.80)	<0.001*
GERD with PPI>1 year(n = 1,345) vs GERD without PPI(n = 38,067)	0.88(0.48, 1.61)	0.675	0.57(0.31, 1.04)	0.066

CI, confidence interval; HR, hazard ratio; PPIs, proton pump inhibitors.

Adjusted for age, sex, hypertension, diabetes mellitus, hyperlipidemia, congestive heart failure, and ischemic stroke.

*P-value < 0.05 was considered statistically significant after test.

## Discussion

To the best of our knowledge, the present study is the largest population-based study in the world to examine GERD as a risk factor for AMI by using a matched cohort design and a long-term follow-up period. The study showed that, in the Taiwan study population, the prevalence rate of GERD was about 5.72%. Some variation in GERD prevalence, ranging from 3.9–25%, has been reported in Taiwan. The difference was usually determined by the used definitions, study patients, and intra- and inter-observer inconsistencies regarding endoscopic diagnosis [12].

Over the last several decades, there has been an increasing awareness of the existing association between GERD and CAD. Some studies have demonstrated that GERD occurs frequently in patients with CAD [8, 9]. However, it remains very difficult to establish whether esophageal reflux can actually provoke myocardial ischemia. In laboratory settings, Mellow and Chauhan have reported that acid perfusion of the esophagus results in decreased coronary blood flow [7, 13]. Davies et al have also shown that instillation of acid into the esophagus can significantly reduce the exertional angina threshold [14]. Several clinical investigators observed an association of gastroesophageal reflux with ECG changes. Lux et al reported that ST changes on Holter monitoring correlated with reflux in 40% of cardiac patient [8]. Dobrzycki et al have also shown that GERD patients have higher incidence of ST depression and a larger total ischemic burden [15]. However, most of the studies have been based on selected populations with only small samples of patients. Only few epidemiological studies are available, and most of them did not include a control group without GERD for comparison. Therefore, the association between GERD and AMI remains unclear. In contrast to our results, one large case-control study has shown no association between GERD and AMI [16].

Our study demonstrated that patients with GERD had significantly higher incidence of AMI than those without GERD in a national population-based cohort. Furthermore, GERD was independently associated with increased risk of developing subsequent AMI. We hypothesize that this relationship may exist through several possible mechanisms. First, the development of AMI after the onset of GERD may be the result of chronic inflammatory process activated by GERD. There are a number of pro-inflammatory mediators (IL-6, IL-8, IL-1β, IFN-γ, TNF-α, reactive oxygen species) produced by the esophageal mucosa which have been shown to be significantly elevated in patients with GERD [17]. Even in non-erosive reflux disease, where an inflammatory role may be considered less obvious, elevated IL-1β and IL-8 levels have also been found [18, 19]. AMI is a multifactorial disease, in which inflammatory processes play an important role [20]. In addition to local inflammation, profound systemic inflammatory response has been documented in AMI patients, which includes elevation of circulating inflammatory cytokines, chemokines, cell adhesion molecules and other immune cells [21]. Elevated IL-6 levels may contribute to the development and instability of atherosclerotic plaques, and high circulating concentrations of IL-6, TNF-α are associated with increased risk of cardiovascular events [22–24]. Second, a vagal visceral reflex between the esophagus and heart has been identified and this allows changes in esophageal function to affect cardiac physiology [4]. Third, GERD and AMI share common risk factors, such as smoking, excess alcohol and obesity [25]. Therefore, it is conceivable that chronic inflammatory process, vagal reflex overstimulation and sharing of common risk factors may be responsible for the association between GERD and AMI.

In the present study, we have analyzed the patients according to whether or not PPIs were administered for GERD therapy. As for the duration use of PPI, we have found that patients who were prescribed PPIs for more than one year had slightly decreased the risk of developing AMI in the future, suggesting that prevention of acidic esophageal mucosal contact may play a role in reducing future myocardial ischemia. Various studies have reported angina suppression as the result of GERD therapy. Liu Y et al. have found that two-week PPI therapy not only reduced cardiac ischemia, but also improved health-related quality of life in patients with CAD and GERD [26]. Dobrzycki and colleagues have shown that short-term PPIs therapy reduced the duration and number of ST-segment depression episodes in patients with GERD and CAD [15]. They concluded that PPIs therapy reduces myocardial ischemia, possibly due to elimination of acid derived esophago-cardiac reflux compromising coronary perfusion. In a large population-based cohort study by Johansson et al [16], they investigated the association between treatment with acid suppressing drugs and the risk of myocardial infarction. No association between treatment and AMI has been observed. The discrepancy could be explained by differences in race and geographic region, but they could also be accounted by how GERD was treated in different studies. The acid suppressing drugs which they used in the UK study included not only PPIs, but also H2-receptor antagonists and antacids. Such inadequate acid control by H2 receptor antagonists or antacids may explain the negative results of the acid-repressing drugs. In Taiwan, the National Health Insurance program covers the use of PPIs only for patients with reflux erosive esophagitis proven by endoscopy. The duration of PPIs use depends on the severity of esophagitis. Non-erosive reflux disease patients who are allowed to use PPIs must have positive esophageal pH-meter monitoring results. However, such methodology is not commonly used in current medical practice in Taiwan. Thus, a greater proportion of GERD patients who received PPIs therapy in our study may have had erosive esophagitis. The differences in the results might be due to different distributions of GERD subgroups. A well-designed prospective randomized study would be needed to compare the effects of erosive and non-erosive reflux disease on PPIs therapy and the risk of AMI.

Additionally, some studies have associated PPI usage with adverse clinical outcomes in high-risk cardiovascular cohorts and general population [27, 28]. Shah et al concluded that PPIs appear to be associated with elevated risk of AMI in the general US population via a data-mining approach in two unrelated datasets [28]. They proposed a possible mechanism by which PPI may promote risk for AMI via an enzymatic pathway resulting in inhibition of nitric oxide synthase and subsequent vasodilatation dysregulation [29]. However, other studies including meta-analyses do not show significant clinical differences in ischemic cardiovascular events or mortality attributed to PPI use [30, 31]. Our study found that patients who were prescribed PPIs for more than one year had slightly decreased the risk of developing AMI in the future. It can be interpreted by the effect of PPIs to prevent of acidic esophageal mucosal contact, and short-term use of PPIs for one year has little adverse cardiovascular effect. To date, the cardiovascular risk from PPI use remains unclear, and needs further research to confirm the risk.

The strength of our study is a matched case-control design using a population based dataset, which provides a large-sized sample of subjects to enable tracing prospectively the association between GERD and AMI, and our adequate controls for comorbidity. However, there are still some limitations in the study. First, the database did not include information on over-the-counter PPI use. In Taiwan, PPIs could be claimed to the NIH only for the patients with erosive esophagitis or peptic ulcer proven by endoscopy. Some patients who refused endoscopic exam bought PPIs from the local pharmacy by themselves. Thus, the effect of PPIs use may have been underestimated. Second, we used ICD-9-CM codes claimed by physicians for GERD without clarifying severity of symptoms as well as endoscopic findings from the database. Therefore, we could not determine the relationship between severity of GERD and AMI. Third, many demographic variables such as socioeconomic status, lifestyle (smoking, alcohol drinking), body mass index, family history of AMI, and treatment compliance are not available in the NHI database.

In conclusion, the present study suggests that GERD may increase the risk of future developing AMI. Further prospective studies are needed to evaluate whether anti-reflux medication such as PPIs may reduce the occurrence of acute ischemic event in GERD patients
